# Current Understanding of the Mechanisms Underlying Immune Evasion From PD-1/PD-L1 Immune Checkpoint Blockade in Head and Neck Cancer

**DOI:** 10.3389/fonc.2020.00268

**Published:** 2020-02-28

**Authors:** Victor C. Kok

**Affiliations:** ^1^Department of Medical Oncology, Kuang Tien General Hospital Cancer Center, Taichung, Taiwan; ^2^Department of Bioinformatics and Medical Engineering, Asia University Taiwan, Taichung, Taiwan

**Keywords:** PD-1/PD-L1 signaling pathway, HNSCC, immune checkpoint blockade, cancer immunotherapy, innate resistance, adapted resistance, immune evasion, head and neck cancer

## Abstract

Starting in 2014, large phase III clinical trials began to disclose the study results of using programmed death (PD)-1 immune checkpoint inhibitors (ICIs) (pembrolizumab, nivolumab) and PD-ligand (L)1 (atezolizumab, durvalumab, avelumab) ICIs immunotherapy in patients with advanced head and neck squamous cell carcinoma (HNSCC). In the recurrent and metastatic (R/M), cisplatin-refractory setting, nivolumab achieved a 2.2-fold increase of the median 1-year overall survival as compared with investigators' choice of salvage chemotherapy (36.0 vs. 16.6%). A paradigm shift to the winning regimen, pembrolizumab combined with platinum and infusional fluorouracil, has outperformed the past gold standard of cetuximab-based platinum and fluorouracil combination in terms of overall survival (median, 13.6 vs. 10.1 mo) when administered as the first-line treatment for R/M HNSCC. Nevertheless, many patients still did not respond to the PD-1/PD-L1 checkpoint inhibitor treatment, indicating innate, adapted, or quickly acquired resistance to the immunotherapy. The mechanisms of resistance to ICIs targeting the PD-1/PD-L1 signaling pathway in the context of HNSCC are the focus of this review. The past 5 years have seen improved understanding of the mechanisms underlying checkpoint inhibition resistance in tumor cells, such as: tumor cell adaption with malfunction of the antigen-presenting machinery via class I human leukocyte antigen (HLA), reintroduction of cyclin D–cyclin-dependent kinase (CDK) 4 complex to cell cycles, enrichment of CD44+ cancer stem-like cells, or development of inactivating mutation in IKZF1 gene; impairment of T-cell functions and proliferation through mutations in the interferon-γ-regulating genes, suppression of the stimulator of interferon genes (STING) pathway, or resulted from constitutional nutritional iron deficiency state; metabolic reprogramming by cancer cells with changes in metabolites such as GTP cyclohydrolase 1, tetrahydrobiopterin, kynurenine, indoleamine 2,3-dioxygenase, and arginase 1; defective dendritic cells, CD-69 sufficient state; and the upregulation or activation of the alternative immune checkpoints, including lymphocyte activation gene-3 (LAG3), T-cell immunoglobulin and ITIM domain (TIGIT)/CD155 pathway, T-cell immunoglobulin mucin-3 (TIM-3), and V domain-containing Ig suppressor of T-cell activation (VISTA). Several potential biomarkers or biosignatures, which could predict the response or resistance to the PD-1/PD-L1 checkpoint immunotherapy, are also discussed.

## Introduction

### Scope of Problems

Head and neck cancers encompass a group of malignancies arising from several anatomical mucosal sites, including the nasal cavity, paranasal sinuses, nasopharynx, oropharynx, hypopharynx, larynx and lips, and oral cavity. According to GLOBOCAN epidemiological estimates of incidence and mortality of cancer worldwide, in 2018, there were ~835,000 new cases of cancer arising from the lips, oral cavity, naso-, oro-, and hypo-pharynx and larynx; with the number of deaths in the same year being ~431,000 (1). The majority (95%) of histopathological types of head and neck cancer is squamous cell carcinoma (HNSCC) (2). Virus-related nasopharyngeal carcinoma will be included in this review because of its immunogenicity and encouraging trial outcomes (3, 4). The tumorigenesis can separate head and neck cancers into virus-related (Epstein-Barr virus-related nasopharyngeal carcinoma (NPC) and human papillomavirus (HPV)-positive oropharyngeal HNSCC) cancer and non-viral (HPV-negative) HNSCC, the latter being related to smoking, alcohol, and betel quid consumption in etiology.

Early-stage and locally advanced stage HNSCC should be treated with curative intent incorporating radical surgical resection or radical radiotherapy combined with chemotherapy. More than 65% of previously treated HNSCC will develop local recurrence or distant metastasis (5). It is very challenging for the head and neck cancer team to manage patients with unresectable locally advanced stage, relapsed or metastatic (R/M) HNSCC, mainly because of the high propensity for intrinsic, spatial, and acquired resistance to chemotherapeutic agents, radiotherapy, and anti-epidermal growth factor receptor monoclonal antibodies. The current first-line regimen for R/M HNSCC adopted a new paradigm in 2019, when the results of the randomized controlled trial, comparing pembrolizumab combined with platinum and infusional 5-fluorouracil to the past gold-standard regimen of cetuximab plus the same chemotherapy combination, confirmed an overall survival benefit (hazard ratio, HR, for death at 0.65, 95% confidence interval, CI, 0.53–0.80) favoring the pembrolizumab-based treatment arm (Table 1). Pembrolizumab is an anti-programmed cell death (PD)-1 monoclonal antibody, previously known as MK-3475, and subsequently, lambrolizumab (8).

**Table 1:** Summary of data demonstrating the evolving new paradigms of systemic treatment for R/M HNSCC over 12 years.

**Outcomes**	**Platinum + 5-FU (6)**	**Cetuximab + Platinum + 5-FU (6)**	Pembrolizumab + Platinum + 5-FU (7)^*^
No. of patients	*n* = 200	*n* = 222	*n* = 281
Overall Response Rate (95% CI)	20% (15–25%)	36% (29–42%)	36.4% (not given)
Progression-Free Survival (mo.)	3.3 (2.9–4.3)	5.6 (5.0–6.0)	HR = 0.84 (95% CI, 0.69–1.02)^*^
Overall Survival in mo. (95% CI)	7.4 (6.4–8.3)	10.1 (8.6–11.2)	13.6 (not given)
Hazard ratio for OS (95% CI)	0.80 (0.64–0.99)		
		0.65 (0.53–0.80)	

* *Results shown here represent the subgroup of patients whose Combined Positive Score (CPS) for PD-L1 was ≥1. The exact figure for progression-free survival was not given in the Abstract*.

### The Rationale of Anti-PD-1/PD-Ligand (L)1 Immunotherapy for R/M HNSCC

One of the hallmarks of cancer is the ability of cancer cells to evade immune destruction (9). PD-1 is a co-inhibitory receptor on the cell surface of cytotoxic T lymphocytes. The ligation of PD-1 and PD-L1 or PD-L2 on tumor cells or antigen-presenting cells (APCs) elicits an immunosuppressive response, which implements subsequent metabolic reprogramming in T cells, decreases effector T cells and memory T cells, and increases Treg and exhausted T cell abundance (10). For the past few years, the abundance of PD-L1 protein in the HNSCC tumor, with its microenvironment sphere, has been the focus of numerous studies (2, 11–17). Observe the oral cavity squamous cell carcinoma (OCSCC) as an example, the prevalence of PD-L1 positivity has been reported in 45-87% of cases, depending on the cut-off value for positivity, whether cytoplasmic staining was counted as positive, and inclusion of the proportion of HPV+ cancer cases (2, 11). The PD-1/PD-L1 axis applies immunosuppressive signals, inducing anergy of cytotoxic T-cells; thus, the blockade of this ligation (analogous to releasing the brake) becomes a strong rationale for anti-PD-1 immunotherapy for R/M HNSCC.

### The Recent Development of Anti-PD-1/PD-L1 Monoclonal Antibody-Based Treatment for R/M HNSCC

Table 2 attempts to summarize the recent relevant clinical trials investigating PD-1 or PD-L1 blockade in R/M HNSCC or NPC. Usually, NPC does not belong to the classical HNSCC membership due to unique tumor pathogenesis and different treatment protocols. However, anti-PD-1 therapy for NPC will be shown in this review to give our readers a broader picture of the immunotherapy comparing classical HNSCCs to NPC. The same would apply to HPV-positive oropharyngeal cancer. Overall response rate (ORR), progression-free survival (PFS), duration of response (DoR), and overall survival (OS) are shown if these results are available in the published paper. To date, only the following two anti-PD-1 monoclonal antibodies, pembrolizumab and nivolumab, and two anti-PD-L1 monoclonal antibodies, durvalumab, and atezolizumab (anti-PD-L1), have been tested in R/M HNSCC (Table 2). As yet, there is only one published phase I study regarding avelumab, a PD-L1 inhibitor, in R/M HNSCC (27–29).

**Table 2:** Summary of clinical trial results of PD-1 or PD-L1 blockade in R/M HNSCC and NPC showing overall response rate (ORR), duration of response (DoR), progression-free survival (PFS), and overall survival (OS).

**1st Author/ published year/ (References)/EudraCT No**.	**Phase of study/Study Name/No. pts**	**Key immunotherapy drug**	**Biomarker**	**Failed treatment previously**	**Treatment outcomes**
Chow/2016/(18)/2012-005771-14	Phase Ib/KEYNOTE-012 Expansion Cohort/*n* = 132	Pembrolizumab at a fixed dose, 200 mg Q3W	Irrespective of biomarker status	57% failed two or more lines of chemo	6-mo. PFS = 23%; 6-mo. OS = 59%. ORR = 22% in PD-L1+ tumors. Duration of response = not reached (range, ≥ 2 to ≥ 11 mo.)
Bauml/2017/(19)/2014-002447-18	Phase II/KEYNOTE-055/*n* = 171	Pembrolizumab	82% PD-L1 positive (CPS ≥ 1%)	75% failed platinum and cetuximab or more	ORR = 16% (95% CI, 11% to 23%). Duration of response = 8 mo. (2+ to 12+ mo.); Median PFS = 2.1 mo., and median OS = 8 mo.
Hsu/2017/(13)/ 2013-004507-39	Phase Ib/KEYNOTE-028/*n* = 27 NPC	Pembrolizumab	Dako 22C3 positive ≥ 1%	70.4% failed three or more lines	ORR = 25.9% (95% CI, 11.1 to 46.3)
Cohen/2019/(20)/2014-001749-26	Phase III/KEYNOTE-040/*n* = 247 (pembro. arm)	Pembrolizumab at a fixed-dose, 200 mg Q3W	PD-L1 tumor proportion score (≥ 50% vs. < 50%)	Failed platinum-containing chemo	ORR = 14·6% (95% CI, 10.4–19.6); Duration of response = 18.4 mo (95% CI 5.8–18.4); Median PFS = 2·1 mo (95% CI 2.1–2.3); Median OS = 8.4 mo. (95% CI 6.4–9.4).
Rischin/2019/(7)/2014-003698-41	Phase III/KEYNOTE-048/*n* = 882 (entire)	Pembrolizumab vs. pembrolizumab + PF vs. cetuximab + PF	CPS for PD-L1 protein expression.	First-line for R/M HNSCC	In the CPS ≥ 1 group, ORR = 36.4% and median OS = 13.6 mo. (in pembrolizumab + PF) vs. ORR, 35.7%; OS, 10.4 mo. (in cetuximab + PF); HR = 0.65, 95% CI, 0.53–0.80).
Ferris/2016/(21);Ferris/2018/(22)/ 2013-003622-86	Phase III/CHECKMATE-141/*n* = 240 (nivo. arm)	Nivolumab 3 mg/kg Q2W	Dako positive ≥ 1%, ≥ 5%, vs. ≥ 10%.	Failed within 6 mo. of platinum therapy	ORR = 13.3% (9.3–18.3); OS = 7.7 mo. (5.7–8.8). 24-mo. OS = 16.9%.
Colevas/2018/(23)/2011-001422-23	Phase Ia/PCD4989g/*n* = 32	Atezolizumab	Responses observed irrespective of HPV or PD-L1 status.	Heavily pretreated	ORR = 22% (95% CI, 9–40%); PFS = 2.6 mo. (0.5–48.4 mo.); Median OS = 6.0 mo (range 0.5–51.6+ mo).
Segal/2019/(24)/not available	Phase I/II expansion/*n* = 62	Durvalumab 10 mg/kg Q2W for 12 mo	32.3% had tumor cell PD-L1 expression ≥ 25%	Failed median of 2 prior systemic treatments (range, 1-13)	ORR = 6.5% (15.0% for PD-L1 ≥ 25%, 2.6% for < 25%); TTP = 2.7 months (range, 1.2-5.5); PFS = 1.4 mo; OS = 8.4 mo. OS rate = 62% at 6 mo and 38% at 12 mo (42% for PD-L1 ≥ 25%, 36% for < 25%).
Siu/2018/(25)/not available	Phase II randomized, open-label/CONDOR/*n* = 67	Durvalumab (10 mg/kg Q2W) monotherapy	PD-L1–low/negative	Failed 1 platinum-containing regimen	ORR = 9.2% (3.46-19.02)
Siu/2018/(25)/not available	Phase II randomized, open-label/ CONDOR/*n* = 133	Durvalumab + tremelimumab (anti-CTLA-4)	PD-L1–low/negative	Failed 1 platinum-containing regimen	ORR = 7.8% (3.78-13.79%)
Bahig/2019/(26)/not available	Phase I-II/*n* = 35 (non-NPC)	Durvalumab (1500 mg Q4W) + tremelimumab (75 mg Q4W × 4 doses) + SBRT to metastases at cycles 2 and 3 of immunotherapy	Biomarker-unselected	Patients with ≥ 2 extracranial metastatic lesions.	Ongoing study
Elbers/2019(27)/not available	Phase I/*n* = 9 (cisplatin-unfit)	Cetuximab-radiotherapy + avelumab (concurrent 10 mg/kg Q2W + 4 months maintenance)	None	Unfit for cisplatin but with an indication for concurrent bioradiotherapy	At 12 (median, 95% CI, 8–26) months follow-up, recurrence occurred in 4/8 patients (50%).
Merlano/2018/(28)/2017-000353-39	Phase Ib-II/CONFRONT/*n* =	Avelumab 10 mg/kg Q2W + Cyclophosphamide 50 mg daily + 8 Gy radiotherapy day 8.	None	Failed at least therapy with platinum, fluorouracil, and cetuximab	Ongoing study.

*EudraCT, European Union Drug Regulating Authorities Clinical Trials; NPC, nasopharyngeal carcinoma; SBRT, stereotactic body radiotherapy; CPS, Combined Positive Score*.

The efficacy of anti-PD-1 and anti-PD-L1 for R/M HNSCC, regardless of its use at as salvage therapy, in the second-line, or even in the first-line while combined with platinum plus infusional 5-fluorouracil, the ORRs were fairly poor across the board. When used in the first-line combined with PF chemotherapy for R/M HNSCC, the response rate was reported as 36.4% (7). Nearly 64% of the patients' tumors demonstrated either primary or adaptive resistance [cancer cells salvaged themselves by resorting to immunoediting and thus, further created an immunosuppressive tumor microenvironment (TME)] (30) to the combination immunochemotherapy.

This review serves to present the recent research findings with implications on the mechanisms of immune evasion from the anti-PD1 or anti-PD-L1 immune checkpoint blockade.

## Methods

### Methodology for the Literature Search

A dynamic PubMed literature search until September 14, 2019, using the Medical Subject Headings and the Boolean search terms, was used to retrieve articles indexed under keywords, such as “head and neck cancer,” “head and neck squamous cell carcinoma,” “HNSCC,” “oral cavity squamous cell carcinoma,” “OCSCC,” “immunotherapy,” “pembrolizumab,” “nivolumab,” “atezolizumab,” “durvalumab,” “avelumab,” “immune escape,” “immune evasion,” “resistance,” “relapsed or metastatic,” “unresected locally advanced,” “mechanism,” “PD-L1,” “PD-1,” “immune checkpoint inhibitor,” “immunoediting,” “tumor microenvironment,” “immunosuppressive,” and “adapted resistance.” The American Society of Clinical Oncology Meeting Abstract database was also searched for the relevant trials. Also, a hand search from the “Similar Articles” inside the PubMed panel was performed to retrieve related articles.

In the EndNote software, “Find Duplicates” function was activated to remove duplicated papers. “Find Full Text” was then activated to download those articles available for download. The Harvard Medical School digital library, “BrowZine,” was then used to complete all full-text downloads for the EndNote library. Review articles represented most retrieved articles in the library, and only a few of them would be cited in this review. Whereas, articles with primary research data from innovative experiments and clinical trials would be selected.

## Results and Discussion

### HNSCC Cancer Cells Remodel and Shape an Immunosuppressive TME

**Figure 1:**
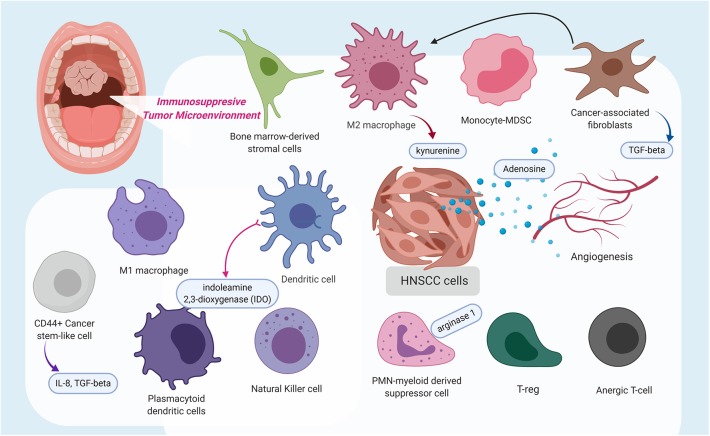
This schematic diagram highlights the immunosuppressive tumor microenvironment (TME) in which a variety of immune cells are polarized to possess pro-tumoral features, stimulated cancer-associated fibroblasts, which release transforming growth factor-β, and even angiogenesis contribute to the immunosuppressive state. Additionally, several molecules, such as kynurenine, adenosine, indoleamine 2,3-dioxygenase, arginase 1, interleukin (IL)-8, and IL-10, were identified to contribute to a pro-tumoral immunosuppressive TME or at extreme, an immune-desert. The figure was created with BioRender.com and was exported under a paid subscription.

Within the tumor microenvironment, there exists a plethora of cytokines, and various infiltrating immune cells, such as CD8+ T cells and APCs, including dendritic cells, anti-tumoral M1 macrophages, pro-tumoral M2 macrophages [tumor-associated macrophages (TAM), myeloid-derived suppressor cells (MDSC), cancer-associated fibroblasts (CAFs), and regulatory T cells (Tregs) (Figure 1)]. Like the immunosuppressive lymphocytic Tregs, MDSC functions as myeloid regulatory cells (MRC) and is further separated into monocytic-MDSC and polymorphonuclear-MDSC (31). A study by Takahashi and colleagues identified that CAFs stimulate and polarize the increase of CD68+ and CD163+ pro-tumoral macrophages in the TME (32).

### Primary Resistance to ICIs in HNSCC

A Birmingham research group developed a 54-gene hypoxia-immune classifier to prognosticate patients with HNSCC, and uncovered that tumors that are high-hypoxia/low-immune are associated with “immune-desert” microenvironmental profiles (33). Hypoxia within a tumor microenvironment will increase the immune-inhibitory PD-L1 generation through the hypoxia-inducible factor (HIF)-1α signaling (33, 34). An immune desert tumor microenvironment promotes immune evasion for cancer cells. These cold tumors respond poorly to single-agent PD-1/PD-L1 immune checkpoint (ICP) immunotherapy. The JAK family of kinases (JAK1/2/3, TYK2) is involved in PD-L1 induction in tumor, stromal, and immune cells (35). A group of investigators from Foundation Medicine Inc., Cambridge, Boston, showed that in a preclinical study focusing on endometrial carcinoma and stomach adenocarcinoma, somatic mutation of JAK1 with loss of function (meaning loss of JAK1-mediated interferon response) results in immune evasion, particularly in microsatellite instability-high (MSI-H) tumors with high total mutational burden (TMB) (35).

Soft tissue infections or deep neck infection in the head and neck region can occur in patients with locally advanced HNSCC. Antibiotic usage around the time of ICI treatment could dampen the effects of PD-1/PD-L1 blockade (36–39), as antibiotics usage for head and neck soft tissue infection will disrupt the integrity of the gut microbiome. A recent study demonstrates that disruptions in the gut microbiome composition, also known as dysbiosis, is one of the mechanisms of primary resistance to ICI therapy (36). Experiments using fecal microbiota transplantation in avatar mice demonstrate that a combination of *A. muciniphila* and *E. hirae* would increase the CCR9- or CXCR3-expressing central memory T cells in the mesenteric lymph nodes and induce dendritic cells to secrete IL-12, a Th1 cytokine essential to elicit immunogenicity during PD-1/PD-L1 blockade (36). However, the actual mechanisms by which the intestinal flora modulate ICP immunotherapy is still unclear.

An immunosuppressive tumor microenvironment can also result from immune modulatory effects of CAF (32), TAM (40), and MDSC (41) (Figure 1). In addition, extracellular signals from angiogenesis can also drive immune suppression by directly suppressing APCs and immune effector cells, or by augmenting the effect of Tregs, MDSC, and TAM. Reciprocally, the above-mentioned immune suppressive cells can also drive angiogenesis (Figure 1), rendering a vicious cycle of disrupted immune activation (42).

### What Does HNSCC Possess to Avoid Immunosurveillance or Destruction via the PD-1/PD-L1 Immune Checkpoint (ICP) Blockade?

To understand the tumor microenvironment of the oral cavity SCC using the infiltrating lymphocyte repertoires as an example, several studies identified considerable antigen-experienced CD4+ and CD8+ cells, Tregs, and PD-1-expressing and Tim-3-expressing T cells, as well as lymphoid follicles with germinal center-like structures (11, 43–48). These immunosuppressive characteristics are indicative of T-cell exhaustion. Table 2 shows that ~60% of patients with R/M HNSCC would not respond to the PD-1/PD-L1 ICP therapy. This means that even the “release of the brakes” through blocking the ligation of PD-1/PD-L1, cannot adequately allow the cytotoxic T cells to mount an attack of significant proportions on cancer cells, which would prevent tumor shrinkage or induce mixed responses amongst measurable tumor sites. The potential mechanisms being recognized for causing acquired immune escape from the PD-1/PD-L1 ICP in the setting of head and neck cancer are summarized in Table 3.

**Table 3:** Mechanisms of immune escape that are implicated in HNSCC.

**Potential mechanisms**	**HPV+ or HPV-**	**Component in the immunity against cancer**	**References**
**TUMOR CELLS ADAPTION**			
Antigen presenting machinery (APM) via class I HLA	No data	Activated CD8+ immunologic pressure could induce transcriptional loss of HLA class one loci; deleterious alterations in *JAK1/2* and β2-microglobulin.	(49–52)
Downregulation of the transporter associated with antigen processing (TAP)-1/2 heterodimer		APM component downregulated by the IFN-γ-phosphorylated STAT1-mediated signaling pathway; results in escaping recognition by tumor antigen-specific cytotoxic T lymphocytes.	(53, 54)
JAK mutation	Both	Leads to loss of sensitivity to IFN-γ signals.	(35)
Cyclin D–CDK4 kinase re-introduction	No data	Destabilizes PD-L1 and controls the PD-L1 abundance in tumor cells.	(55)
Enrichment of CD44+ cancer stem-like cells	No data	Activates immunosuppressive network through cytokine release.	(54)
IKZF1-inactivating mutations	No data	Genomic alterations of the master regulator IKZF1 correlates with low immune recruitment.	(56)
**IMPAIRED T-CELL FUNCTIONS AND PROLIFERATION**			
Nutritional iron deficiency state	Both	Affects T-cell proliferation	(57–59)
Mutations in interferon-γ-regulating genes	Both	Exhausted “Immune Class” enriched with M2 macrophages, WNT/TGF-β activation	(60, 61)
Suppression of stimulator of interferon genes (STING) pathway	HPV+	Dampens the antitumor immune response.	(62)
Inhibition of STAT1 phosphorylation	Both	Enhanced T-cell exhaustion and accumulation of MDSCs	(63)
**CHANGES IN METABOLITE- AND CYTOKINE-RICH TUMOR MICROENVIRONMENTS**			
Defective dendritic cells (DC)	Both	Defective cytokine- and STAT-mediated regulation of DC.	(64–66)
CD69-sufficient state	Both	Leads to effector T-cell exhaustion.	(67)
Genetic inactivation of GTP cyclohydrolase 1 (GCP1)	No data	Drastically impairs T-cell maturation.	(58)
Metabolite tetrahydrobiopterin (BH4) inhibited by kynurenine	No data	Will impair T cell function.	(58)
Indoleamine 2,3-dioxygenase-1 (IDO1); Tryptophan metabolite, kynurenine (Kyn) level	Both	IDO1 inhibits T cell proliferation, restricts tumor immune infiltration, and retards antitumor immune responses. Kyn released from ϕ, and myeloid cells activate T-reg cells.	(12, 68, 69)
Cancer-associated fibroblasts secrete TGF-β	Both	Results in restraining CD8+ T effector cells infiltrating into microenvironment. TGF-β1 also decreases the number of dendritic cells in the draining lymph nodes.	(32, 70–73)
Arginase 1 expression on microenvironment myeloid cells	Both	Arg1 leads to L-arginine depletion depriving T cells and NK cells of essential nutrients required for proliferation.	(74)
CD38-upregulation	Both	CD38 inhibits CD8+ T-cell function via adenosine receptor signaling.	(75, 76)
Ectonucleotidases CD39/CD73 axis	Both	CD39 is considered a tumor-specific dysfunction marker. Tregs use the axis to diminish anti-cancer killing.	(76–79)
Polymorphonuclear myeloid-derived suppressor cells (PMN-MDSC) activation	Both	Through the nitric oxide pathway, PMN-MDSCs impair proliferation and expression molecules in activated T cells.	(41)
Nucleotide-binding domain leucine-rich repeat and pyrin domain containing receptor 3 (NLRP3) inflammasome activation	Both	Leads to downstream interleukin (IL)-1β release. NLRP3 inflammasome/IL-1β axis increases MDSCs, Tregs and TAMs creating an immunosuppressive microenvironment.	(80)
**ACTIVATION OF AND DEPENDENCE ON ALTERNATIVE IMMUNE CHECKPOINTS**			
Lymphocyte activation gene-3 (LAG3) (=CD223) upregulation	More in HPV+	Induces a state of functional exhaustion in effector T-cells.	(81, 82)
T-cell immunoglobulin and ITIM domain (TIGIT)/CD155 pathway activation	Both	Augments TIGIT+ T-regs, a unique T-reg subset, leading to active suppression of anti-tumor immune response and T-cell exhaustion.	(82–85)
T-cell immunoglobulin mucin-3 (TIM-3) upregulation	Both	TIM-3 is considered a tumor-specific dysfunction marker. It dampens effector T-cell functions in the microenvironment.	(44, 48, 77, 86)
V domain-containing Ig suppressor of T-cell activation (VISTA)	Both	Leads to T-cell exhaustion and T-reg recruitment in the microenvironment.	(87, 88)

*Investigated mechanisms of acquired immune escape from PD-1/PD-L1 checkpoint blockade relevant to head and neck cancer*.

There are currently four areas of research on the molecular resistance mechanisms underlying the selective pressure from PD-1/PD-L1 ICB (Table 3). In this review, they are categorized as tumor cell adaption, impairment of T cell functions and proliferation, changes in metabolite- and cytokine-rich tumor microenvironments, and activation of and dependence on alternative immune checkpoints.

### Tumor Cell Adaption

Tumor cell adaption occurs through various processes, namely with modification of molecules causing immune escape via malfunction of the antigen-presenting machinery via class I HLA, reintroduction of cyclin D–CDK4 kinase heterodimers to cell cycles (55), enrichment of CD44+ cancer stem-like cells (54), JAK mutation (35), or development of inactivating mutation in IKZF1 gene, the master regulator of immune infiltrates recruitment (56).

Speckle-type POZ protein (SPOP) is an E3 ubiquitin ligase adaptor protein mediating poly-ubiquitination and proteasome-mediated degradation of several proteins, such as PD-L1 (55, 89). SPOP mutations or copy number variation can act as a tumor suppressor or progressor depending on the different context in different cancer types. In head and neck cancer, TCGA dataset, <2% of patient tumors exhibited mutated or altered SPOP, which was associated with non-significant reduction of the relative risk of relapse or disease progression (relative risk = 0.4, 95% confidence interval, 0.06–2.61) (Supplementary Table 1). Recent research identified that cyclin D-CDK4 kinase reintroduction in CAF would destabilize PD-L1 molecules via the cullin 3-SPOP pathway (55). Whereas, loss of function mutations of SPOP will increase the level of PD-L1 and tumor-infiltrating lymphocytes, as observed in mouse models. CDK4/6 inhibitor treatment will theoretically increase PD-L1 levels thus potentiating the ICB treatment (55).

In the milieu of the HNSCC immune microenvironment (Figure 1), there exists a kind of CD44+ stem-like cell, whose immunosuppressive capabilities have been demonstrated to be more effective than CD44-negative stem cells to inhibit the effector T-cell population while simultaneously inducing immunosuppressive Tregs and MDSC (54).

Genes function as a master regulator to control the expression of downstream genes by controlling transcription factors. IKZF1 is such a master regulator that could lead to enhanced recruitment of immune infiltrate and tumor sensitivity to ICP inhibitors in several cancer types. Genomic alterations of this gene could negatively affect immunogenicity and tumor response to ICB (56).

Results from genomic studies of HNSCC demonstrate that the immune evasion genetic pathways are different between HPV-negative and HPV-positive HNSCCs (24). The HLA mutations are uncommonly found with <10% prevalence rate in HPV-negative HNSCCs, whereas, the HPV-positive tumors will have more common HLA and Beta2-microglobulin (B2M) mutations and TRAF3 loss (90). B2M is a light chain incorporated with the MHC Class I heavy chain to form a capable antigen-presenting machinery complex. B2M deficiency in tumor cells, which results in defective cell surface HLA Class I complex or subsequent acquired loss of B2M while under immunotherapy, has been recognized as a crucial immune escape mechanism as demonstrated in various solid tumor models (91).

### Impairment in T Cell Functions and Proliferation

Potential mechanisms of intrinsic or adaptive resistance rest upon T-cell functions and proliferation, which is the most essential weapon the human body utilizes to destroy cancer cells. T-cell functions and proliferation can be impaired through mutations in interferon-γ-regulating genes, suppression of the Stimulator of Interferon genes (STING) pathway or result from constitutional nutritional iron deficiency states (57–59, 62). It is noted that HPV-antigen expression levels in the tumor microenvironment would enhance cytotoxic T lymphocyte dysregulation (77).

### Changes in Metabolite- and Cytokine-Rich Tumor Microenvironments

The other well-known hallmark of cancer is metabolic reprogramming by cancer cells. The tumor extracellular microenvironment contains a vibrant display of metabolites and cytokines released from different cell types aiming to create an immunosuppressive environment for the proliferation of tumor cells. Table 3 highlights several notable changes in metabolite-rich and cytokine-rich tumor stroma with consequences of dampening anti-cancer immunity. Defective cytokine- and STAT-mediated regulation of dendritic cells (DCs) leads to defective DCs. CD-69 sufficient state will cause effector T-cell exhaustion. The actual mechanism is still elusive; however, it is apparent that CD-69 expressed on leucocytes is responsible for cell retention in the tumor microenvironment and the CD-69 expression on T-cells is associated with the expression of PD-1 and Tim-3 in T-cells (67).

The function of metabolites GTP cyclohydrolase 1 (GCH1) and tetrahydrobiopterin (BH4) has been identified to be able to increase T-cell proliferation and promote their maturation. GCH1 is the first rate-limiting enzyme in the *de novo* BH4-synthesis pathway. Whereas, metabolite kynurenine has been found to activate Treg cells, and the enzyme indoleamine 2,3-dioxygenase (IDO) inhibits T cell proliferation and restricts tumor infiltration. Arginase 1 (Arg1) depletes L-arginine, depriving the essential nutrients that T cells and NK cells need to proliferate. The alterations of these metabolites will adversely impact anti-cancer immunity through various molecular mechanisms.

### Activation of and Dependence on Alternative Immune Checkpoints

The fourth group of mechanisms underlying the resistance to PD-1-PD-L1 blockade includes activation of and dependence on alternative immune checkpoints. These alternative immune checkpoints other than PD-L1 include lymphocyte activation gene-3 (LAG3) (=CD223) upregulation, T-cell immunoglobulin and ITIM domain (TIGIT)/CD155 pathway activation, T-cell immunoglobulin mucin-3 (TIM-3) upregulation, and V domain-containing Ig suppressor of T-cell activation (VISTA). The past two decades have seen research identify the G-protein-coupled adenosine receptors mediating downregulation of the inflammatory tumor microenvironment creating an immunosuppressive milieu. Adenosine and adenosine triphosphate (ATP) are the most abundant metabolites within a cell and in the extracellular space, which acts as an autocrine and paracrine messenger. While ATP acts as an accelerator to promote proinflammatory activities, adenosine, via the Gs-coupled A2a and A2b receptors, suppresses various immune cells, including T lymphocytes, NK cells, neutrophils, dendritic cells, and macrophages (75, 76, 78, 92–95).

There are several vital molecules interplaying within the *adenosine receptor signaling*. The molecule, CD73, is an ecto-5-nucleotidase, which will dephosphorylate extracellular AMP to immunosuppressive adenosine (78, 79, 94). As a ubiquitous membranous ectozyme, CD38, cleaves NAD(+) and NADP(+), generating cyclic ADP ribose (cADPR), NAADP, and ADPR, which are directly involved in the calcium signaling essential for a cell (96). Current evidence suggests that one of the significant mechanisms in acquired immune escape from PD-1/PD-L1 inhibition is CD38 upregulation. CD38 promotes adenosine production through the **CD38-CD203a-CD73 axis**. Studies discovered that the tumor cells hijacked and leveraged the adenosine receptor signaling pathway by upregulating the activities of CD38, to develop resistance to PD-1/PD-L1 immunotherapy through inhibition of CD8+ T-cell function. Upregulation of CD38 is induced by tumor-derived soluble mediators, all-trans retinoic acid (ATRA) and IFNβ, in the tumor microenvironment.

### Prediction of Response to Anti-PD-1 Immunotherapy in HNSCC

The development of predictive precision oncology aiming to discover and validate biomarkers that can predict the response from PD-1/PD-L1 immunotherapy has evolved relentlessly in the past years. Table 4 highlights the current discovery of biomarker predictors for immunotherapy response in patients who received PD-1/PD-L1 ICB. The eagerly awaited issues to be solved in predictive biomarker development are to achieve adequate clinical evidence about the discriminative capacity (power) of each biomarker. While the evidence is being investigated through clinical studies, Table 4 aims to demonstrate some potentially investigated or helpful biomarkers related to the field of HNSCC. These include the combined positive score (CPS) for PD-L1 protein expression, mismatch repair (MMR)-deficient status, apolipoprotein B mRNA editing enzyme, catalytic polypeptide-like (APOBEC)-driven mutations status, the molecular exhausted immune class, molecular active immune class, the interferon-γ signature (6-genes), the expanded immune signature (18-genes), the condition of somatic frameshift events in tumor suppressor genes, the total mutational burden (TMB), and the microenvironment infiltrating arginase 1 (Arg1)+/CD68+ macrophage-mediated immune evasion state.

**Table 4:** Response prediction to anti-PD-1 immunotherapy in HNSCC.

**Gene alterations or signature**	**Testing platform**	**Response to anti-PD-1 checkpoint blockade**	**References**
Combined Positive Score (CPS) for PD-L1 protein Expression	Immunohistochemistry on formalin-fixed paraffin-embedded tissue samples	CPS = number of PD-L1+ tumor cells, lymphocytes, and macrophages, divided by the total number of viable tumor cells, and multiplying by 100. In various trials, CPS ≥ 1 predicts response.	(7, 19, 97, 98)
MMR-deficient	Quantification of genomic MSI level (MSI intensity)	Higher insertion-deletion (Indel) load predicts response.	(99)
Apolipoprotein B mRNA editing enzyme, catalytic polypeptide-like (APOBEC)-driven mutations	APOBEC enrichment scores.	Upregulated as an innate immune response particularly in HPV+ tumors. APOBEC3 mutation leads to driver mutation in *PI3KCA*.	(100–102)
Molecular exhausted immune class	Gene expression pattern analyzed by non-negative matrix factorization algorithm	Portends a worse prognosis than active immune class in overall survival.	(61)
Molecular active immune class		Better prognosis (overall survival) than exhausted class. May predict immune responses.	(61)
Interferon-γ signature (6-genes)	NanoString nCounter mRNA	Low signature score did not respond to pembrolizumab.	(103)
Expanded immune signature (18-genes)	NanoString nCounter mRNA	Low signature score did not respond to pembrolizumab.	(103)
Somatic frameshift events in tumor suppressor genes	Targeted massively parallel sequencing	More frequently seen in HPV- responders.	(104, 105)
Total mutational burden (TMB)	Targeted massively parallel sequencing	Predicts response in HPV- HNSCC.	(104, 105)
Microenvironment infiltrating arginase 1 (Arg1)+/CD68+ macrophage-mediated immune evasion	Enzyme-Linked Immunosorbent Assay (ELISA)	Plasma Arg1 level (ng/mL) to predict immune evasion (cutoff to be determined)	(74)

The immunohistochemistry combined positive score (CPS) for quantifying the PD-L1 expression in a tumor sample has been adopted to predict tumor response to ICB treatment. This score is calculated as the number of PD-L1+ tumor cells, lymphocytes, and macrophages, divided by the total number of viable tumor cells, and multiplying by 100. In various trials, CPS ≥ 1% predicts immunotherapy response. The caveat of using CPS at the 1% cutoff to select HNSCC patients for PD-1 ICB is that intratumor heterogeineity should be considered. Rasmussen et al. have prospectively investigated the intratumor heterogeneity in PD-L1 expression in HNSCC in 28 patients with a total of 33 whole surgical specimens (98). With 1% cut off, 52% of the six random core biopsies from each surgical specimen was concordant with CPS. Defining a tumor as positive if just a single-one of the core biopsies was positive using CPS at 1% cut-off, the negative predictive value of a single negative core biopsy was 0% (98).

DNA mismatch repair (MMR) genes, hMLH1 and hMSH2, once inactivated, can accumulate thousands of mutations in simple repeats in genomic DNA and develop microsatellite instability (MSI). A defective MMR system results in both MSI and high tumor mutation burden (TMB-high), which further generates neoantigens to be identified by APCs to mount an effective cytotoxic killing of tumor cells. It has been estimated that MSI is present in ~40% of patients with HNSCC (106). The frequency of MSI in an endemic betel-quid chewing region is about the same as the otherwise non-endemic regions, having a rate of 37.9% (107). Despite the fact that these TMB-high cancers are more immunogenic, unlike those immunogenic-desert tumors, half of the TMB-high patients still do not respond to anti-PD-1 immunotherapy (108). Subsequently, a study looking at the mutational landscape of MSI-high discovered that the extent of immunotherapy response is specifically associated with a mutational load accumulating the insertion-deletion (indel) mutations (99). The authors emphasize that there is a greater impact of frameshifting indel mutations over the more general missense mutations, in eliciting anti-PD-1 immunotherapy response.

Generally speaking, TMB was defined as the total number of somatic, coding, base substitution, and indel mutations counted per megabase (Mb) of genome interrogated (109). In terms of the mutational burden as assessed by real-time gene sequencing, HNSCC exhibits median mutations/Mb of 5.0 with 10.1% (95% CI, 8.5%−11.9%) having > 20 mutations/Mb (109). In a group of 81 patients with HNSCC, higher TMB, as demonstrated by the targeted massively parallel tumor sequencing, predicted PD-1/PD-L1 ICB response (*P* < 0.01) (105).

### T Cell-Inflamed Gene Signature

IFN-γ signaling (expanded immune) signature consists of the following 18 genes: *CD3D, CCL5, CD3D, CD3E, IL2RG, CIITA, GZMK, CXCL9, CXCR6, TAGAP, CD2, HLA-E, IDO1, LAG3, NKG7, GZMB, STAT1*, and *CXCL10* (60). Typically, the core IFN-γ gene signature comprises six genes, i.e., *IDO1, CXCL9, CXCL10, HLA-DRA, STAT1*, and *IFNG* (60). In a retrospective analysis of the correlation of the 18-gene signature score (as a continuous variable) with the best of response (BOR) and PFS for 43 patients with HNSCC of the KEYNOTE-012 cohort (18, 110), strong statistical significance (*P* = 0.015 and *P* < 0.001, respectively) was obtained. The 18-gene IFN-γ signature profile derived from the NanoString platform is under development as a clinical-grade companion diagnostic incorporated into the future or ongoing pembrolizumab trials (111).

An enriched proinflammatory M1 macrophage signature, enhanced cytolytic activity, abundant tumor-infiltrating lymphocytes, high human papillomavirus (HPV) infection, and favorable prognosis were associated with active immune class (all, *P* < 0.05).

A bioinformatics study using a non-negative matrix factorization algorithm of the RNA sequencing profiles of 522 patients with HNSCC collected in the TCGA identified an immune class (61). This immune class was determined based on the enriched inflammatory response, enhanced cytolytic activity, and active interferon-γ signaling. There are two subclasses within the immune class, namely, the exhausted immune class, with a poor prognosis, and the active immune class, with a favorable prognosis. The active immune class was highlighted to have enriched anti-tumoral M1 macrophage polarization, stronger cytolytic activity, abundant tumor-infiltrating lymphocytes, and higher HPV infection rates (61). The research findings are relevant to the clinical application on selecting patients with active immune signatures for immunotherapy.

Finally, IFN-gamma signaling depends on the integrity of the JAK/STAT pathway. An orthotopic head and neck squamous cell carcinoma model experiment using Stat1 deficient [Stat1(–/–)] mice observed enhanced T-cell exhaustion and accumulation of MDSCs, creating a tumor-permissive microenvironment in Stat1 deficient mice (63).

Based on current research findings, if tumor samples are discovered possessing any of the aforementioned genetic aberrations or signatures indicating a hot immune tumor, a treatment recommendation incorporating the blockade of the PD-1/PD-L1 axis is justified. The status of recommendation on selecting a predictive biomarker will be refined when new evidence or data is available to suggest otherwise.

## Conclusions

Hnsccs have more immunosuppressive tumor microenvironments although the initial research indicated that HNSCCs are amongst the top malignancies ranked from high to low by the neoantigen loads and total tumor burdens. Although a new treatment paradigm has been established placing pembrolizumab + platinum and infusional fluorouracil as the new standard as the first line for R/M HNSCC, ~64% of patients will not benefit from the significant tumor regression criteria, indicating innate, adaptive resistance to the blockade of PD-1/PD-L1 ligation. This review assimilated the research findings after an extensive literature review and presents the potential mechanisms of immune escape from the PD-1/PD-L1 checkpoint inhibition into four aspects as follows: tumor cell adaption, impairment of T-cell functions and proliferation, changes in metabolite- and cytokine-rich tumor microenvironments, and activation of the alternative immune checkpoints. There are no easy methods for immunotherapy response prediction in HNSCC. Even the widely accepted criteria of the combined positivity score for PD-L1 protein expression, commonly adopted in several clinical trials to stratify patients with HNSCC by the degree of immunogenicity, may have a negative predictive value of zero percent if just one core-biopsy was examined, questioning the usefulness of this immunohistochemistry test in unresectable patients. Targeted massively parallel sequencing and NanoString nCounter mRNA analysis showing the results of total mutational burden or certain immune signatures present promising tests with the potential to discriminate between immune responsive or unresponsive patients with HNSCC, thus requiring further research to confirm their utility in predictive precision immuno-oncology. Every patient with HNSCC contemplating ICI therapy, theoretically, should be considered for several tests, namely TMB, MSI, PD-L1/L2 genome amplification, viral antigens, and next-generation sequencing, to identify actionable targets to combine with ICI therapy.

## Author Contributions

The sole author of this work performed all aspects related to a review article, such as research idea conception, research strategy planning, literature search, retrieval and selection, extensive analysis of data, drafting the manuscript, drawing figures, creating tables, and finally approved this manuscript for publishing.

### Conflict of Interest

The author declares that the research was conducted in the absence of any commercial or financial relationships that could be construed as a potential conflict of interest.

## Abbreviations

APOBEC: Apolipoprotein B mRNA editing enzyme, catalytic polypeptide-like
BDSC: Bone marrow-derived stromal cell
BMSC: Bone marrow-derived suppressor cell
CPS: Combined positive score
HNSCC: Head and neck squamous cell carcinoma
ICB: Immune checkpoint blockade
ICP: Immune checkpoint
IDO1: Indoleamine 2,3-dioxygenase-1
IKZF1: IKAROS family zinc finger 1
JAK: Janus kinase
MDSC: Myeloid-derived suppressor cell
MMR: Mismatched Repair
ORR: Overall Response Rate
PD-L1: Programmed death-ligand 1
PFS: Progression-free survival
R/M: Relapsed/metastatic
STAT: signal transducer and activator of transcription
STING: Stimulator of Interferon Genes
TCGA: The Cancer Genome Atlas (program)
TIGIT: T-cell immunoglobulin and ITIM domain
Tim-3: T cell immunoglobulin and mucin-domain containing-3
TMB: Tumor mutation burden
TME: Tumor microenvironment
VISTA: V domain-containing Ig suppressor of T-cell activation.
