# Supplementary material for: Current Understanding of the Mechanisms Underlying Immune Evasion From PD-1/PD-L1 Immune Checkpoint Blockade in Head and Neck Cancer
Source: Front Oncol. 2020 Feb 28;10:268. doi: 10.3389/fonc.2020.00268 (PMC7058818; doi:10.3389/fonc.2020.00268)
Supplement: Supplementary file 1 [file Table_1.docx]

**Current Understanding of the Mechanisms Underlying Immune Evasion From PD-1/PD-L1 Immune Checkpoint Blockade in Head and Neck Cancer**

Victor C. Kok

**Supplementary Table 1.**

| Supplementary Table 1. Genomic mutation in *SPOP* in HNSCC tumor appears associated with fewer, 60% less, relapse or disease progression. | | |
| --- | --- | --- |
|  | With Relapsed/Progression | Relapse-free/Progression-free |
| *SPOP* mutated or altered | 1 | 4 |
| *SPOP* wild type | 168 | 264 |
|  | Relative risk = 0.40 (95% CI = 0.06–2.61) | |
| Across TCGA HNSCC cohorts and three independent cohorts from Broad Institute, Johns Hopkins, and MD Anderson, 437 tumors underwent pre-treatment sequencing of *SPOP* gene. | | |
